# Ernest Blacker

**Published:** 1911-03

**Authors:** 


### ERNEST BLACKER, M.D.Durh., L.R.C.P.Ed., M.R.C.S.Eng.

1853–1911.

THE uniqueness of individuals is a striking scientific fact, but it is just one of those facts that can hardly be done justice to in an obituary notice.

In writing this brief memoir of my departed friend, Ernest Blacker, I am regretfully conscious of this shortcoming.

It may, however, be stated that his was a cheery, neat, alert and debonair personality, rich in human sympathies and sociabilities. Consequently his interests in life were manifold and varied.

It is no doubt true of modern humanity that its fundamental conceptions of life and destiny tend to run into scientific or anti-scientific grooves, and in this momentous matter Blacker's predilections were all for science. For him science was not merely an accompaniment or condition of human progress, but human progress itself.

Not that he had much sympathy with that form of science which, seated on Olympus, wrapped in the snowy mantle of Detachment, contemplates the flux of life in its manifold permutations and combinations with the impassivity of the Sphinx.

No; it was rather with the form of science which focuses all its energies on social amelioration that Blacker sympathised. He hoped that in some degree the sufferings of the world might thus be abated. And he was conscious that these were not man's alone, for his sympathies went out in a marked degree to the animal world.

In the last decade of his life he was much engrossed with the problem of diet reform, seeking in the vegetable rather than in the animal world the chief requirements of subsistence.

Ernest Blacker was born at Midsomer Norton, where his father was a life-long medical practitioner.

He received his professional education at the Bristol medical establishments, and by his assiduity in his student days gained the much desired prize for practical anatomy in 1876.

In 1878 he obtained by examination the diplomas of L.R.C.P.Edin. and M.R.C.S.Eng.

After some varied professional experiences, he succeeded to his father's practice at Midsomer Norton, and during this time he was medical officer to the Clutton Union Workhouse.

After many years' service in this field of activity he migrated to Clifton, where he became partner in his brother's practice.

In 1896 he graduated as M.D. at the University of Durham.

Subsequently he practised at Southampton and at Farnborough, and finally settled at 8 Meridian Road, Redland.

Although for the last few years of his life he had retired from general practice, he was generally occupied with professional duties as *locum tenens*, until incapacitated by the malady to which he succumbed, after a year's illness. He may thus be said to have died in harness.

Some twelve years ago Dr. Blacker married Miss Edith Harvey, of Clifton, who survives him.

His illness was borne with braveness and resignation, sustained by the devoted attentions of wife and sister.

It was on January 29th that he died, and he was buried at Midsomer Norton on February 4th.

